# Increased Natural Killer Cell Activation in HIV-Infected Immunologic Non-Responders Correlates with CD4+ T Cell Recovery after Antiretroviral Therapy and Viral Suppression

**DOI:** 10.1371/journal.pone.0167640

**Published:** 2017-01-11

**Authors:** Zhenwu Luo, Zhen Li, Lisa Martin, Zhiliang Hu, Hao Wu, Zhuang Wan, Michael Kilby, Sonya L. Heath, Lei Huang, Wei Jiang

**Affiliations:** 1 Department of Microbiology and Immunology, Medical University of South Carolina, Charleston, United States of America; 2 Beijing You’an Hospital, Capital Medical University, No.8 Xitoutiao, You’an men wai, Fengtai District, Beijing, China; 3 Divison of Infectious Diseases, Department of Medicine, Medical University of South Carolina, Charleston, United States of America; 4 Department of Infectious Disease, the Second Affiliated Hospital of the Southeast University, Nanjing, China; 5 Division of Infectious Diseases, Department of Medicine, University of Alabama at Birmingham, Birmingham, AL, United States of America; 6 The 302 Hospital of PLA, Treatment and Research Center for Infectious Diseases, Beijing, China; Karolinska Institutet Department of Medicine Solna, SWEDEN

## Abstract

The role of natural killer (NK) cell function in HIV disease especially in the setting of long-term antiretroviral therapy (ART) and viral suppression is not fully understood. In the current study, we have investigated NK cell activation in healthy controls and aviremic ART-treated HIV+ subjects with different degrees of immune restoration. We performed a cross sectional study in 12 healthy controls and 24 aviremic ART-treated HIV-infected subjects including 13 HIV+ subjects with CD4+ T cells above 500 cells/μL defined as “immunologic responders” and 11 HIV+ subjects with CD4+ T cells below 350 cells/μL defined as “immunologic non-responders”. We analyzed NK cell number, subset, and activation by expression of CD107a and NKG2D and co-expression of CD38 and HLA-DR. NK cell-mediated cytotoxicity against uninfected CD4+ T cells was tested *in vitro*. We found that NK cell absolute number, percentage of NK cells, and percentage of NK cell subsets were similar in the three study groups. The increased NK cell activation was found predominantly in CD56dimCD16+ subset of immunologic non-responders but not immunologic responders compared to healthy controls. The activation of NK cells was inversely correlated with the peripheral CD4+ T cell count in HIV+ subjects, even after controlling for chronic T cell activation, sex, and age, potential contributors for CD4+ T cell counts in HIV disease. Interestingly, NK cells from immunologic non-responders mediated cytotoxicity against uninfected CD4+ T cells *ex vivo*. NK cells may play a role in blunted CD4+ T cell recovery in ART-treated HIV disease.

## Introduction

Antiretroviral therapy (ART) dramatically suppresses HIV viral replication, improves immune function, restores CD4+ T cells, increases survival, and delays disease progression in HIV disease [1–3]. However, up to 25% patients fail to reconstitute their CD4+ T cells to the levels similar to healthy controls despite HIV suppression under ART treatment [2, 4, 5]. Inflammatory syndrome, heightened morbidity and mortality, are seen in persons who fail to increase their CD4+ T cell counts under ART treatment [3, 6–10]. Patients under viral-suppressive ART treatment with peripheral CD4+ T cell counts > 500 cells/μl are defined as “immunologic responders” and patients under viral-suppressive ART treatment with peripheral CD4+ T cell counts ≤ 350 cells/μl are defined as “immunologic non-responders” [11–13]. The mechanisms of incomplete CD4+ T cell restoration have been extensively studied, including thymic and lymphoid fibrosis, low nadir CD4+ T cell counts, heightened microbial translocation and inflammation, high T cell activation, and virus-mediated cytopathogenicity [14–23]; but are still not fully understood.

Chronic T cell activation is a predictive marker for peripheral CD4+ T cell count and disease progression in HIV disease [24–26]. Although ART treatment significantly decreases chronic T cell activation, residual activation persists even after many years of ART treatment [27, 28]. The magnitude of T cell activation in this setting is associated with the degrees of CD4+ T cell recovery [29].

Natural killer (NK) cells are a subset of granular lymphocytes that are critical in the innate immunity against infection [30]. They are capable to kill infected cells through cytolysis [31]. Moreover, NK cell mediated antibody-dependent cellular cytotoxicity (ADCC) has been associated with protection from infection and disease progression [32], and is an important mechanism to control HIV infection [33–35]. Human NK cells are commonly defined to two subsets, CD56dimCD16+ and CD56brightCD16− subpopulations [36]. CD56dimCD16+ NK cells predominate in the peripheral blood, and CD56brightCD16− NK cells constitute the majority of NK cells in secondary lymphoid tissues [36]. Chronic HIV infection is associated with reduced proportion and absolute number of CD3−CD56+ NK cells compared to healthy controls [37, 38], as well as increased proportion of CD56dimCD16+ NK cell subset [39, 40]. These alterations of NK cells are largely associated with HIV viral replication in ART-naïve patients [41].

Several markers are associated with NK cell activation and cytolytic function. For example, co-expression of CD38 and HLA-DR is an activation marker on NK cells [42]. CD107a is a marker of lysosomal granule exocytosis [43]. Upon antibody-mediated NK cell activation, CD107a expression increases and mediates cytolytic activity [43, 44]. NKG2D is evolutionary conserved and encoded in human chromosome 12 within the NK cell gene complex [45]. NKG2D has been shown to regulate NK cell cytotoxicity and cytokine production [46]. Notably, increased levels of NK cell activation and functional markers have been reported in HIV disease and are largely controlled by ART treatment [42, 47]. For example, the percentages of IFN-γ, TNF-α, and CD107a-expressing NK cells were significantly higher in ART-naïve patients but were similar in treated patients compared to healthy controls [43]. Moreover, NK cells express Fc receptor CD16, which can bind to IgG1 and IgG3 subclasses [30]. Fc of HIV specific antibodies binds to its receptors on HIV infected cells and triggers cell lysis through Fc receptor on NK cells [31]. However, NK cell cytolysis was impaired and was associated with HIV disease progression [48, 49]. Notably, chronic immune activation has a significant impact on NK cell dysfunction in HIV disease [50]. Nonetheless, the role of NK cells in ART-treated HIV-infected patients with poor CD4+ T cell recovery is not fully understood.

To better understand the role of NK cells in CD4+ T cell recovery after ART treatment, we examined and analyzed NK cell activation in healthy controls, HIV+ immunologic non-responders and HIV+ immunologic responders. We found that NK cells were activated predominantly in CD56dimCD16− subset in immunologic non-responders compared to responders and healthy controls. The activation of NK cells was inversely correlated with peripheral CD4+ T cell counts even after controlling for confounding factors.

## Methods

### Study subjects

This study was approved by the Institutional Review Board for Human Research (IRB) at the Medical University of South Carolina. All participants were adult ages above 20 and provided written consent. In the current study, 12 healthy controls and 24 HIV+ ART-treated aviremic HIV-infected subjects were evaluated in a cross sectional study. The clinical characteristics of healthy controls, 13 immunologic responders, and 11 immunologic non-responders are shown in Table 1 and S1 Table.

**Table 1:** Clinical characteristics.

	Healthy control	HIV+/IR	HIV+/INR	P value (IR vs INR)
Total no. of subjects	12	13	11	
No. of male/female	3 ⁄ 9	11 ⁄ 2	6 ⁄ 5	0.08
Age (yr)	44 (34–58)	43 (31–49)	47 (36–56)	0.21
Plasma HIV RNA		Not detectable	Not detectable	
CD4+ T cell counts	782 (513–944)	733 (658–777)	322 (225–349)	< 0.0001
Years of ART		15 (13.8–15.0)	14 (11.0–16.0)	0.46
%CD38+HLA-DR+CD4+		1.17 (1.0–2.2)	1.96 (1.2–3.2)	0.09
Nadir CD4+ T cell counts		320 (215–507)	170 (92–195)	0.001

CD4^+^ T cell counts (cells/μl).

Plasma HIV RNA (copies/ml).

Data are medians (interquartile ranges).

### Flow cytometry

Peripheral blood mononuclear cells (PBMC) were isolated over a Ficoll-Hypaque cushion (GE, Pittsburgh, PA) from EDTA-contained blood, aliquot, and stored at -80°C before use. Antibodies were incubated with PBMC at 4°C for 30 min for surface staining. After surface staining, the cells were washed and analyzed by flow cytometry. The following fluorochrome-labeled monoclonal antibodies were used (clone): anti-human CD3-percp (OKT3), anti-CD56-APC (B-159), anti-CD16-PEcy7 (3G8), anti-human CD4-BV421 (RPA-T4), anti-human CD107a-BV500 (H4A3), anti-human CD38-FITC (HIT2), anti-human HLA-DR-PE (G46-6), anti-human NKG2D-BV500 (1D11), and Ghost Red 780 (Tonbo Biosciences, San Diego, CA). Mouse IgG1-BV500, IgG1-APC and IgG2a-PE isotype antibodies were used to gate on CD107a/NKG2D-BV500, CD38 and HLA-DR respectively. Cells were collected by BD FACSVerse Flow Cytometer (BD Biosciences) and data were analyzed by FlowJo software (Version 10.0.8).

### NK cell-mediated CD4+ T cell cytotoxicity

NK cell-mediated CD4+ T cell cytotoxicity was assessed by flow cytometry. Briefly, purified CD4+ T cells from the same healthy control donor were co-cultured with or without purified NK cells at a 1:3 ratio in 96-well V-bottom plates (Corning). Cells were incubated for 15 min at room temperature, spun at 300 g for 1 min, and incubated for 6 h at 37°C. After incubation, cells were surface stained with antibodies against CD3, and fixed with 2% paraformaldehyde solution containing a constant number of flow cytometry particles (5*10^4^/ml) (AccuCount blank particles, 5.3 μm; Spherotech, Lake Forest, IL). A constant number of particles (2.5*10^3^) were counted during cytometry acquisition in order to normalize the number of CD4+ T cells. The percentage of CD4+ T cell cytolysis was calculated using the formula: %cytolysis = [(number of CD3+ T cells in the absence of NK cells)–(number of CD3+ T cells in the presence of NK cells)]/(number of CD3+ T cells in the absence of NK cells) *100.

### Statistical analysis

All data were analyzed and graphed using Prism software (GraphPad 6.0) and SPSS (Version 23). Statistical significance between two groups was determined by the Mann-Whitney test (non-parametric). In the pre-specified hypothesis, we were interested in the comparisons of HIV+ immunologic non-responders versus HIV+ immunologic responders or healthy controls; therefore, p-values from comparing HIV+ immunologic non-responders to each of control groups were not adjusted for multiple comparisons [51]. Associations between pairs of continuous variables were analyzed by Spearman Correlation test. Partial correlation (controlling for age, sex, and frequency of CD38+ on CD4+ T cells) between CD4+ T cell counts and NK cell activation was analyzed using SPSS (Version 23).

## Results

### NK cell number and subsets in healthy controls, HIV-infected immunologic responders, and HIV-infected immunologic non-responders

**Fig 1:**
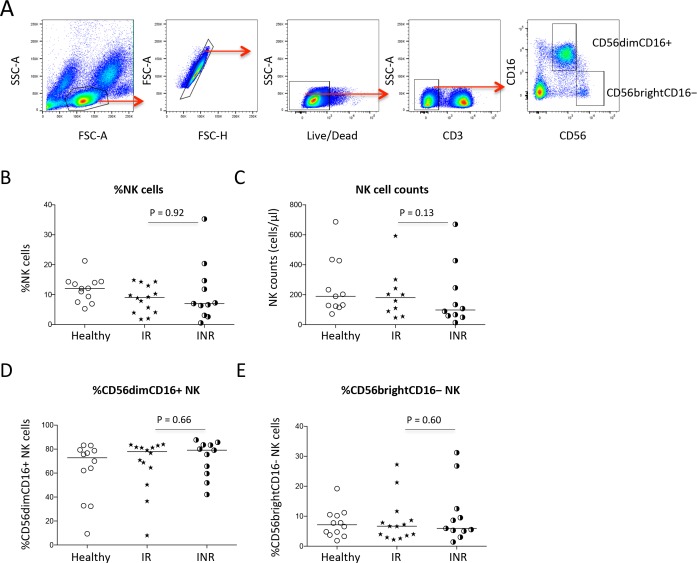
NK cells and subsets distribution. **Blood samples were stained with fluorescent-labeled antibodies and tested by flow cytometry**. (A) Representative dot plots, showing the gating strategies used to assess NK cells, and CD56dimCD16+ or CD56brightCD16− NK cell subsets. The median frequency (B) and absolute count (C) of NK cells were shown in immunologic non-responders, immunologic responders, and healthy controls. The median frequencies of CD56dimCD16+ (D) or CD56brightCD16− subsets (E) in NK cells were shown in the three study groups.

To study NK cells in the setting of HIV+ subjects with different degrees of immune restoration, the absolute cell count and percentages of NK cell and subset (CD56dimCD16+ and CD56brightCD16−) were measured in 12 healthy controls, 11 HIV+ immunologic responders and 13 HIV+ immunologic non-responders patients under viral-suppressive ART treatment. Consistent with previously studies [40], NK cell counts and the percentages of NK cell in PBMC tended to be lower in immunologic non-responders compare to immunologic responders and healthy controls, but the differences did not achieve statistical significance (Fig 1A–1C). The percentages of CD56dimCD16+ and CD56brightCD16− NK cell subpopulations were also similar in the three study groups (Fig 1D and 1E).

### NK cell activation, CD107a, and NKG2D expression in HIV-infected immunologic non-responders

**Fig 2:**
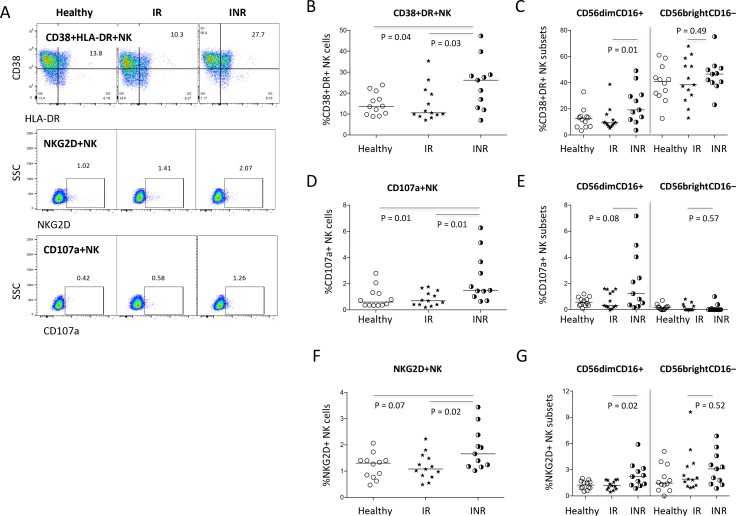
NK cell activation in treated HIV disease. (A) Dot plots represent the gating strategies from one representative donor of each study group. The median frequencies of CD38+HLA-DR+ NK cells (B) and NK cell subsets (C) among healthy controls, immunologic responders, and immunologic non-responders. The median frequencies of CD107a+ on NK cells (D) and NK cell subsets (E) in healthy controls, immunologic responders, and immunologic non-responders. The median frequencies of NKG2D+ on NK cells (F) and NK cell subsets (G) in healthy controls, immunologic responders, and immunologic non-responders.

To further determine NK cell activation in HIV+ subjects with different degrees of immune restoration after long-term ART treatment and viral suppression, we analyzed the expression of CD38, HLA-DR, CD107a, and NKG2D on NK cells in the current study. In the current study, co-expression of CD38 and HLA-DR on NK cells in all ART-treated patients was similar compared to healthy controls (P = 0.68, Fig 2A and 2B). However, if HIV+ subjects were stratified into immunologic responders and non-responders, the non-responders had significant higher frequency of CD38 and HLA-DR co-expression compared to responders and healthy controls (Fig 2A and 2B). As expected, CD38+HLA-DR+ co-expression on CD56dimCD16+ NK subset was significantly increased in immunologic non-responders compared to the other two study groups (p < 0.05, Fig 2C). The other NK cell subset, CD56brightCD16−, without expression of the IgG receptor FcRγIIIA (CD16), had similar levels of CD38 and HLA-DR co-expression in the three study groups (P > 0.05, Fig 2C).

To analyze NK cell parameters related to cytotoxicity, we have assessed surface CD107a and NKG2D expression on NK cells. CD107a expression on NK cells was elevated in immunologic non-responders compared to immunologic responders and healthy controls (Fig 2D, p < 0.05), while CD107a expression in NK cells was similar in immunologic responders and healthy controls (Fig 2D). Notably, CD107a-expressing NK cells were predominantly in CD56dimCD16+ NK cells but not in CD56brightCD16− NK cells (Fig 2E), suggesting that cytolytic function is enriched in CD56dimCD16+ NK cell subset. Moreover, the frequency of surface NKG2D-expressing NK cells increased in NK cells, especially in the CD56dimCD16+ subset among immunologic non-responders compare to immunologic responders (Fig 2F and 2G); there was no difference of CD107a and NKG2D expression in CD56brightCD16- NK cells among the three study groups (Fig 2E and 2G). These results suggest that CD56dimCD16+ NK cell subset are activated and may have cytolytic function *in vivo* in aviremic ART-treated HIV+ subjects with incomplete CD4+ T cell recovery.

### Correlations of CD38 and HLA-DR co-expression, CD107a, and NKG2D expression on NK cells

**Fig 3:**
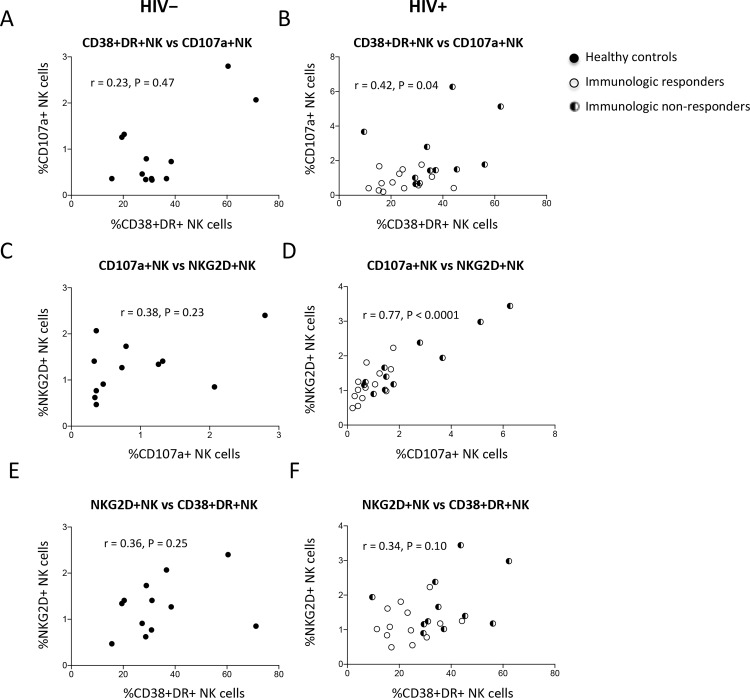
Correlations of NK cell activation in healthy controls and ART-treated HIV disease. Correlations between the percentages of CD107a-expressing NK cells and co-expression of CD38 and HLA-DR on NK cells in healthy controls (A) and HIV+ subjects (B), between the percentages of CD107a-expressing NK cells and NKG2D-expressing NK cells in healthy controls (C) and HIV+ subjects (D), and between the percentages of NKG2D-expressing NK cells and co-expression of CD38 and HLA-DR on NK cells in healthy controls (E) and HIV+ subjects (F).

Next, we analyzed the correlations between activation and functional markers on NK cells in healthy controls and HIV+ subjects (Fig 3A–3F). Interestingly, no correlation was found in healthy controls (Fig 3A, 3C, and 3E), but there were direct correlations between the percentages of CD107a-expressing NK cells and co-expression of CD38 and HLA-DR on NK cells (Fig 3B), and between the percentages of CD107a-expressing NK cells and NKG2D-expressing NK cells (Fig 3D) in all HIV+ subjects. The correlation between the percentages of NKG2D-expressing NK cells and co-expression of CD38 and HLA-DR on NK cells in HIV+ subjects tended to correlate, however did not achieve significant difference (Fig 3F). These results suggest that NK cells may be activated to express these activation and functional markers by different mechanisms in healthy individuals but by a similar mechanism in treated HIV-infected patients.

### Correlation between NK cell activation and CD4+ T cell counts

**Fig 4:**
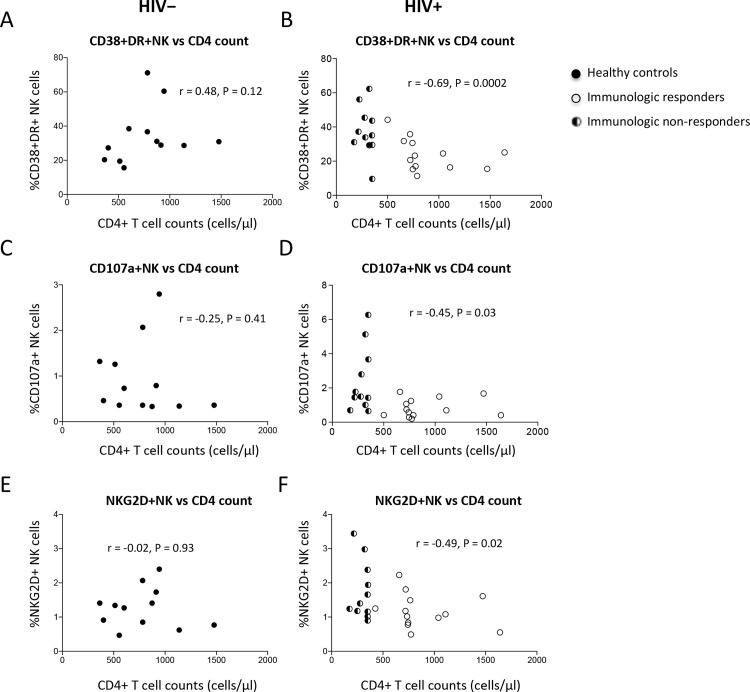
NK cell activation and peripheral CD4+ T cell counts. Correlations between the percentages of co-expression of CD38 and HLA-DR on NK cells and CD4+ T cell counts in healthy controls (A) and HIV+ subjects (B), between the percentages of CD107a-expressing NK cells and CD4+ T cell counts in healthy controls (C) and HIV+ subjects (D), and between the percentages of NKG2D-expressing NK cells and CD4+ T cell counts in healthy controls (E) and HIV+ subjects (F).

To investigate NK cells in HIV disease, we analyze the correlation between NK cell activation and CD4+ T cell reconstitution after long-term ART treatment and viral suppression in HIV+ subjects. Notably, NK cell activation and function reflected by co-expression of CD38 and HLA-DR, and expression of CD107a and NKG2D, were all inversely correlated with peripheral CD4+ T cell counts in HIV+ subjects, but not in healthy controls (Fig 4). It is well know that chronic T cell activation contributes to CD4+ T cell depletion in chronic HIV infection [52, 53], and sex and age are associated with CD4+ T cell counts [54, 55]; we therefore have analyzed the inverse correlation between NK activation and CD4+ T cell counts after controlling these potential contributors. Interestingly, the correlation between CD4+ T cell count and the percentage of CD38 and HLA-DR co-expression on NK cells (r = -0.48, P = 0.03) was still significant, but neither the correlation between CD4+ T cell count and the percentage of CD107a-expressing (r = -0.34, P = 0.20) nor the correlation between CD4+ T cell count and NKG2D-expressing (r = -0.40, P = 0.13) NK cells was significant in HIV+ subjects after controlling of age, sex, and CD4+ T cell activation. These results suggest that long-term ART treatment did not fully normalize NK cell activation, and NK cell activation is associated with CD4+ T cell reconstitution.

### Correlations between NK cell subset activation and CD4+ T cell counts in viral-suppressed and ART-treated HIV+ subjects

To determine which NK cell subpopulation is associated with CD4+ T cell recovery, we analyzed the correlations between the percentages of activation in NK cell subsets and CD4+ T cell counts (Table 2). Interestingly, we found that the frequency of NKG2D expression and CD38 and HLA-DR co-expression on CD56dimCD16+ NK cells was significantly correlated with CD4+ T cell counts (Table 2). Furthermore, after controlling of CD4+ T cell activation, sex and age, the correlation between CD4+ T cell counts and co-expression of CD38 and HLA-DR on CD56dimCD16+ NK cell subsets was still significant (r = -0.48, P = 0.03), but the correlation between CD4+ T cell counts and NKG2D (r = -0.31, P = 0.18) or CD107a (r = -0.23, P = 0.33) expression in CD56dimCD16+ NK cell subsets was not significant in HIV+ subjects. Together these data reveal that the activation and function of CD56dimCD16+ NK cell subset were associated with CD4+ T cell recovery in viral-suppressed and long-term ART-treated HIV disease.

**Table 2:** Correlations between NK cell subset activation and CD4+ T cell counts in HIV+ subjects.

NK subset activation	%CD107a+ NK	%NKG2D+ NK	%CD38+DR+NK
CD4 count vs CD56dimCD16+	r = -0.28	r = -0.48	r = -0.66
	P = 0.17	P = 0.02	P < 0.0001
CD4 count vs CD56brightCD16−	r = 0.08	r = -0.23	r = -0.33
	P = 0.63	P = 0.18	P = 0.05

r: correlation coefficient.

P: P value.

### Cytotoxicity of NK cells from immunologic non-responders

**Fig 5:**
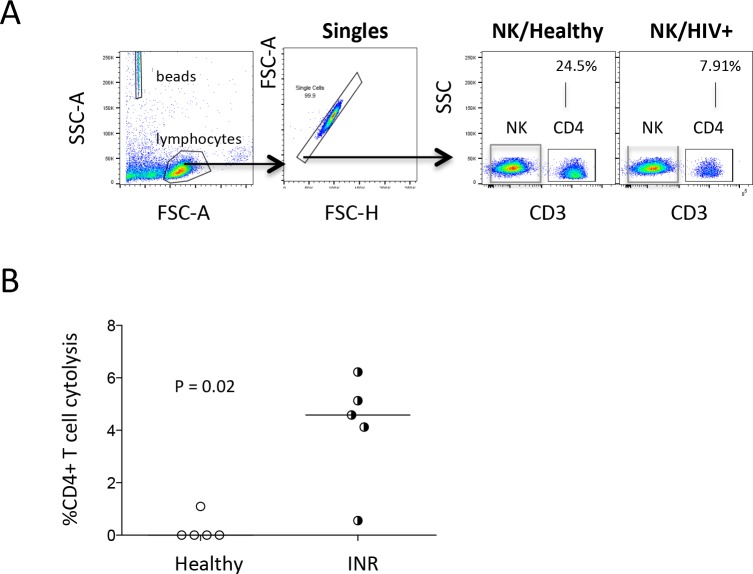
NK cells from immunologic non-responders mediate CD4+ T cell death *in vitro*. NK cells were isolated from 5 healthy controls and 5 ART-treated viral-suppressed immunologic non-responders. CD4+ T cells were isolated from the same healthy donor. CD4+ T cells were cultured with isolated NK cells at a ratio of 1:3. The percentage of CD4+ T cell cytolysis was analyzed. (A) Dot plots were shown from a representative healthy control donor and a representative HIV+ donor. (B) The median percentages of CD4+ T cell cytolysis were shown with co-cultured NK cells from healthy controls and immunologic non-responders.

Cytotoxic or activated NK cells have been found to accumulate in lymph nodes after HIV/SIV infection [56, 57]. To determine whether NK cells from HIV+ immune non-responders were activated to gain the function of cytotoxicity against CD4+ T cells, we have isolated NK cells ex vivo and co-cultured with CD4+ T cells from the same healthy control donor. NK cell-mediated cytotoxicity was assessed by flow cytometry. Notably, activated NK cells have been shown to express CD4 [58], we thus used CD3 antibody to stain CD4+ T cells in the co-cultured system containing NK cells and CD4+ T cells to avoid miscalculation of CD4+ T cells. Interestingly, NK cells from immunologic non-responders but not from healthy controls had the ability to induce CD4+ T cell death (Fig 5), suggesting that NK cells from immunologic non-responders may be activated and cytotoxic to induce uninfected CD4+ T cell death in immunologic non-responders *in vivo*. These results suggest that NK cells may play a role in CD4+ T cell depletion in HIV disease.

## Discussion

In this study, we found that long-term ART treatment and viral suppression controls NK cell activation in HIV-infected immunologic responders to the level similar to healthy controls. However, HIV-infected immunologic non-responders still exhibit elevated NK cell activation, and the magnitude of NK cell activation, CD107a, and NKG2D expression were inversely related to CD4+ T cell counts in HIV+ subjects.

The results on the role of NK cell function in HIV disease are conflicting [31, 33, 35, 42, 47, 59]. NK cells are shown to control HIV replication and kill HIV-infected cells through ADCC [60]. On the other hand, NK cells have been shown to associate with HIV disease pathogenesis. For example, in untreated HIV disease, NK cell activation was correlated with CD4+ T cell counts, plasma level of HIV RNA, T cell activation, plasma sCD14 and inflammation [41, 42]. However, in another study in ART-treated and untreated patients, neither plasma level of HIV RNA nor CD4+ T cell counts was correlated with NK cell activation [47]. Notably, NK cells from long-term non-progressors who naturally control viremia have greater NK cell function compared to progressors and healthy controls [61, 62]. Nonetheless, NK cell function is impaired during HIV infection [33]. The possible mechanisms include the followings: 1) HIV virus induces activation and dysfunction of NK cells; 2) decreased levels of perforin and granzyme A in NK cells may account for impaired NK cytotoxicity [63]; 3) NK cells are activated *in vivo* in HIV disease (Figs 2 and 5), therefore they are desensitized to be re-stimulated and function as ADCC *in vitro*; 4) DC and NK cell cross-talk is dysregulated through IL-12/IL-15 during HIV infection [64, 65]; and 5) changes in NK cell subsets in untreated HIV disease may play a role in their function. Most studies on NK cell dysfunction were among high viremia and untreated patients [38, 42]. In the current study, we focus on the role of NK cells in CD4+ T cell recovery among ART-treated and viral-suppressed HIV-infected patients.

CD56brightCD16− NK cell subset is the majority of NK cells in lymph nodes [36]; they do not have strong cytotoxic activity but produce cytokines such as IFN-γ and TNF-α upon activation [30]. They may be precursors of CD56dimCD16+ NK cells [36], previously research shows that NK cells in HIV-1-infected patients from Ugandans display elevated activation, and low CD4+ T cell counts were associated with increased levels of IFN-γ and degranulation in CD56bright NK cells [66]. CD56dimCD16+ NK cell subset is the predominant NK cell subset in the periphery; they express FcγR IIIa (CD16) and enable NK cell cytotoxicity [30]. In HIV/SIV infection, NK cells have impaired function of cytotoxicity and IFN-γ production [59, 61]. Notably, CD56dimCD16+ subset has been shown to play a role in HIV pathogenesis [61]. Consistently, we found that levels of NK cell activation and functional markers increased in CD56dimCD16+ NK cells from immunologic non-responders but not responders compared to healthy controls, and that NK cell activation and functional markers in CD56dimCD16+ NK cells was inversely correlated with CD4+ T cell counts in aviremic ART-treated HIV+ subjects.

NK cell activation has been correlated with plasma level of soluble CD14, a factor associated with microbial translocation in one study [42], but not in another study [47]. The drivers of NK cell activation in immunologic non-responders in viral-suppressed HIV disease are not known. The virus still actively replicates in lymph nodes [67–69] in patients (most likely immunologic non-responders) even after long-term plasma viral-suppressive ART treatment [70–72], thus the virus may play a role in NK cell activation in lymph nodes. Furthermore, antibodies may contribute to NK cell activation due to their ability to activate NK cells via Fc receptor and mediate cell death through NK cell cytotoxicity [30]. Altogether, the residual active HIV replication in lymph nodes, inflammation, or antibodies may play a role in NK cell activation among immunologic non-responders in treated HIV disease.

The mechanisms of blunted CD4+ T cell restoration after long-term viral-suppressive ART treatment is not fully understood. Thymic output is measured by the T cell receptor excision circle (TREC) assay, but results are inconclusive [73–77]. Increased T cell activation and expansion result in decreased TREC numbers, which has been linked to increased T cell activation and expansion rather than impaired thymic output in HIV [77, 78]. Other factors such as sustained increases in microbial translocation and inflammation, low nadir and pre-ART naïve CD4+ T cell counts, and high T cell activation, have been associated with CD4+ T cell recovery post-ART treatment [14, 52, 79–84]. In the current study, we found that markers of NK cell activation and function were inversely related to CD4+ T cell counts even after controlling of T cell activation, sex, and age. Isolated NK cells from immunologic non-responders induced CD4+ T cell death from the healthy donor *in vitro*. Our results reveal a novel mechanism that NK cell activation may play a role in blunted CD4+ T cell recovery in ART-treated HIV disease.

## Supporting Information

S1 Table Clinical characteristics of participants. (PDF)
