# Supplementary material for: Increased Natural Killer Cell Activation in HIV-Infected Immunologic Non-Responders Correlates with CD4+ T Cell Recovery after Antiretroviral Therapy and Viral Suppression
Source: PLoS One. 2017 Jan 11;12(1):e0167640. doi: 10.1371/journal.pone.0167640 (PMC5226712; doi:10.1371/journal.pone.0167640)
Supplement: S1 Table — (PDF) [file pone.0167640.s001.pdf]

	CD4count	nadirCD4	CD38+DR+CD4	age	Yr of ART
INR	350	195	0.95	36	26
INR	349	361	3.2	26	4
INR	348	92	2.35	47	13
INR	350	234	1.79	61	14
INR	216	12	1.24	36	17
INR	174	155	2.91	47	16
INR	276	66	1.96	66	15
INR	283	180	1.12	54	15
INR	225	168	7.68	45	12
INR	322	170	1.18	51	3
INR	322	176	10.8	47	11
IR	788	216		28	15
IR	658	306	1	32	15
IR	697	681	9.79	45	15
IR	431	334	1.17	55	15
IR	722	187	1.04	55	33
IR	1109	1137	1.1	43	5
IR	743	239	2.16	25	15
IR	658	23	1.1	43	15
IR	720	282	2.24	36	15
IR	773	213	1.59	43	3
IR	426	467	2.22	22	13
IR	766	473	0.56	48	14
IR	1641	506	0.61	40	27
IR	746	509	1.6	51	15
Healthy	513			58	
Healthy	601			49	
Healthy	914			38	
Healthy	401			28	
Healthy	554			49	
Healthy	1479			38	
Healthy	782			35	
Healthy	944			58	
Healthy	782			58	
Healthy	874			33	
Healthy	1138			57	
Healthy	363			32	
